# Prophylaxis

**Published:** 1892-03

**Authors:** J. Taft


### Prophylaxis.

BY J. TAFT.

Read before the Ohio State Dental Society, held at Columbus, December, 1891.

Oral hygiene is a subject which every dentist perhaps imagines he understands quite well. It is a subject, however, that receives but little consideration in the literature of the profession, or in our discussions upon dental questions, and altogether too little in the actual practice of the average dentist. It is because of these facts that I now ask your attention briefly to this subject.

The general failure to recognize the practical phase of this subject, is not because of a corresponding want of knowledge or want of ability to meet the emergencies, but it is due in a large measure to the neglect of the subject in our literature and discussions. There is a very great lack of understanding and appreciation of the importance of this subject by the people generally, and so it is rare indeed to find any one with a mouth with all its parts in a perfect state of health and purity; notwithstanding the fact, that upon the condition of the mouth and teeth, in a large measure, depend the comfort and welfare of the entire organism.

The process of digestion and nutrition, in a large measuer, depend upon the insalivation and thorough mastication of the food. This can only be accomplished when the masticators are in good condition, and this can only be maintained by intelligent care and attention ; and the latter will only be exercised when there is a proper appreciation of the value of these organs.

Perhaps not more than one in twenty, in any ordinary community, succeeds in maintaining the mouth and teeth in a good state of health and purity. This does not occur so much from absolute ignorance as to how it can be accomplished, as from want of appreciation of the importance of the subject ; for it is quite practicable, and within the reach of every one who desires to maintain these organs in the best possible state of health, comfort and usefulness, to secure a very great improvement over the average condition as presented.

And now, are there any others to whom responsibility attaches for this state of affairs ? Can it be said that physicians, who are supposed to be the conservators of the public health, are much more appreciative than the common people ?

Very seldom indeed is it that physicians give instructions, to those in their charge, about the health and preservation of their teeth. The truth is, that usually the chief efforts of the physician are directed to the cure of disease, rather than to its prevention ; notwithstanding, the latter is vastly more important than the former.

May we ask how much care is exercised in respect to this matter, by physicians, each one for himself?

The want of attention to the subject of both special and general hygiene, on the part of the medical profession, is lamentably prevalent, to it vastly more attention should be given.

What shall be said in regard to the attitude of dentists in regard to oral hygiene? Are they not relatively as much in fault in this matter, as the general physician? How seldom is it that dentists are careful to instruct, and sufficiently impress upon the minds of those they may have in charge, the importance, the benefit, and the means of maintaining a good state of health of the mouth and teeth.

Is it not true that in a large proportion of cases, and may we not say a large majority, in which the dentists are called upon for remedial and restorative treatment, that they wholly overlook prophylaxis? How often is it that the operation of filling teeth is performed in a mouth having much disease, and a large amount of debris and impurities that ought to be in the first place removed.

How often is it that the dentist simply rests satisfied with performing an operation for repair upon a defective tooth or teeth, when the first thing that should receive attention, is a restoration to health and purity of the mouth in all its particulars, and the operations upon decayed teeth for restoration, should follow.

It will readily be conceded that when a patient is placed in charge of a dentist, for the care and preservation of the teeth, it is his bounden duty to do whatever will minister to the best interest of that patient; and this applies not only in respect to restoration, repair and remedy, but also to prevention as well; the proverb, "Prevention is better than cure," applies here with great force.

While it is true that the active and manipulative work of the dentist is, in a large measure, devoted to repair and cure, yet he has, if possible, a greater responsibility and duty in aiding and directing his patient in such a course of health and habit, as shall most certainly ward off and secure him against disease.

Is it not true that patients, many a time, are dismissed from the care of the dentist, with the mouth in such a condition with respect to impurity, that the best work possible upon the teeth in the way of filling, is of but little permanent value? Every patient before being dismissed should receive such instruction, and be so impressed with the importance of the subject, that the habit of attention to the care of the mouth should be thoroughly established, and as persistently followed as that of sleep, or partaking of food. The dentist owes it to himself and his profession, as well as to his patient, to do whatever will lead to the greatest permanency and security of his operations.

No one at this day should be permitted to engage in the practice of dentistry without thorough knowledge and the best appreciation of dental hygiene, and all that pertains to it. Every dental college should make oral hygiene one of the most important of the branches of its instruction. This subject should be more discussed in dental societies than it has been, and with a special reference to arousing attention in regard to it; this is perhaps more necessary than giving instruction as to methods of procedure; nevertheless, the latter is very important, and more should be written upon the subject.

In looking over the literature of dentistry there is but little found on this subject, a few brief articles, probably not over half a dozen, will be found in our periodical literature bearing directly on this subject, and few if any of these assume to discuss the question to any good degree of thoroughness. There has been enough written to fill volumes on "The Management of Pulpless Teeth"; "Abscessed Teeth"; ,,Supplying Artificial Substitutes," etc. ; while the subject of oral hygiene has received but a word or a thought at long intervals. Our textual literature is well nigh silent on the subject of the maintenance of the mouth and teeth in a state of health; they deal almost wholly with processes of remedy, repair and restoration. This deficiency does not occur wholly from ignorance, for nearly all dentists, and many others, know full well the ordinary results of the neglect of the teeth and mouth. Time and space forbid that we here enter into a full discussion of oral hygiene. Is it not quite as necessary that we apply the knowledge we have of this subject, as to strive for more knowledge while we are not utilizing what we have? I doubt not that three-fourths of the diseases of the mouth and teeth are preventable by a right application and exercise of the knowledge we have upon the subject of prophylaxis. There is, of course, much to be learned, and it is commendable to make all possible progress, but let us, by all means, appreciate and apply the knowledge and ability we have; if we possessed more light and knowledge, and made no better use of it than of the knowledge we already have, we would be none the better, but worse.

In the further consideration of this subject, I will ask your attention to one or two offensive conditions of the teeth, with which all dentists are more or less familiar; all are familiar with the ordinary deposits and accretions that are found in the neglected mouth.

Perhaps the most simple and least excusable of these is a more or less vitiated mass of soft pasty matter, consisting of food material, thickened mucus, and waste from the adjacent tissues. This is usually found in greatest amount at and near the necks of the teeth, and beneath the margins of the gums, and between the teeth ; this material ordinarily undergoes putrefactive change quite rapidly. The character of this change, and the form which the material may assume, will be modified by the state of fluids of the mouth, by the character of the breath, and somewhat doubtless, by the condition of the alimentary canal ; the change in this material will also be influenced by "mouth breathing" ; this evil habit tends to produce thickening of the mucus of the mouth by evaporation of its water ; variation in thermal changes is also by this means induced ; foreign matter in the form of dust organisms, and whatever may be in the air, will be taken into the mouth by the " mouth breather." All this tends to hasten change of matter that may be lodged in the mouth ; this variety of accumulation may be found upon all the teeth in the mouth, perhaps in larger amount, however, upon the buccal surfaces of the molars, and the anterior surfaces of the inferior incisors, and oftentimes upon the labial of the superior incisors and cuspids, upon the latter it is usually found in its most offensive condition ; upon the entire surface of any or all of the teeth this accumulation will often be found, and especially is this true of those not used in mastication. It will usually be found in its most offensive state where fresh saliva comes most in contact with it, and so, nowhere is it found more offensive than upon the labial surfaces of the superior anterior teeth. The influence of this material is injurious in several aspects or directions ; it usually possesses a more or less marked offensive odor, due to its putrefaction, and this is often given to the breath ; and in " mouth breathers" this offensive condition will be carried to the lungs, as well as thrown out by the breath. This offensive matter also necessarily more or less mixes with the food, and is taken into the alimentary track ; this accumulation is usually more or less irritating to the margin of the gums, and to the mucous membrane.

In all cases where it exists in any considerable quantity at and beneath the margin of the gum, there will be an inflamed condition of the tissue, more or less pronounced, and of greater or less outreach according to the susceptibility of the tissues, and in many cases persistent suppuration of the margin of the gums is the result, and sometimes disease thus induced extends into the sockets of the teeth, causing more or less pronounced disease there.

The persistent presence of this material especially upon the buccal surface of the superior molars, and upon the labial surface of the superior incisors, is a fruitful source of decay of these teeth, and especially so where there may be defective enamel, or where it passes beneath the margins of the gum and beyond the edge of the enamel; evidence of this destructive influence is shown by the fact that very rarely does decay occur upon this surface of these teeth when they have been kept wholly free from this deposit.

This accumulation is almost universally found upon the teeth of those who do not masticate as nature designed. Those who live upon pultaceous food, requiring little or no friction in the act of mastication, usually have this material in large quantities. Different kinds of food vary in their liability to undergo change; some under a given influence will decompose rapidly, others under the same influence much less rapidly.

In giving directions for the hygienic management of the mouth, the things here referred to must be taken into consideration, viz: the habit of mouth breathing has its deleterious effects upon the mouth, the teeth, the throat, etc. The patient should be made to understand the influence of a vitiated breath, and to understand that there is always more or less of mischief in connection with it that ought to be remedied. Always when an offensive breath is present, it should be a matter of earnest solicitude on the part of the patient; the condition of the alimentary canal oftimes has much to do in vitiating the breath, and in inducing an abnormal state or condition of affairs, but a course of life should be adopted by the patient such as will change conditions for the better.

"The mouth breather" should contract the habit of keeping the mouth closed, and using the legitimate organ for respiration; the best possible condition of the general system should be maintained by a full observance of the laws of health and hygiene, to this end, good and nutritious food should be used, mastication and insalivation should be thoroughly accomplished, all the eliminating organs of the body should be maintained in a most perfect condition, each one should be so managed and treated that its peculiar work will be well accomplished. To these conditions very little, if any attention is given, by the average practitioner of dentistry. The remark was not long since made, in the hearing of the writer, by a prominent dentist, that "he did not regard it as his duty, nor the business of a dentist, to cleanse the teeth from that which the patient himself could remove." Now, while in one sense this may be true, it does betray a want of appreciation in respect to this matter; it is as important that the teeth and mouth receive attention in regard to purity and cleanliness, as that decayed teeth be filled, or that diseased gums, or alveolar abscess be treated.

A great many excuses are made for neglect in this matter, such as: "I have been sick, or not very well, and so my teeth have been neglected." Another, "I have been away from home and had not the opportunity to attend to my teeth"; "I have not the appliances or preparations for keeping my teeth in good condition"; "I did not know that there was anything objectionable or pernicious about or upon my teeth, or even in the mouth." Another says, "I have been so very busy that I had not time to give attention to this matter." How much influence any or all of these excuses ought to have, or will have with any one who fully appreciates the value or importance of a healthy mouth and good teeth, I leave you to decide.

Let us then, as we go forth in the exercise of our professional duties, use most efficiently the knowledge and resources we have. In time of peace prepare for war; in time of health build barriers against the incursion of disease, and not wait till it is upon us, and then put forth efforts in meeting it, which are in so many instances fruitless, and at best serve but a very temporary purpose.

---

DISCUSSION.

*Dr. C. P. Dennis*, Portsmouth. This subject is too important to permit it to go by without some discussion, as it is one we have to deal with in our daily practice. I will undertake to say that it is much like our religious duties, there is so much difference between the profession and our conduct in the matter. It is a very important matter also because the durability of our operations in the mouth depend so largely upon the condition of the mouth and teeth and the care that is taken of them. I try to impress upon every patient who comes into my office the necessity for caring for the teeth, and when I have not got time to talk about it, I give them printed directions. It seems to me, we cannot expect operations to be lasting unless the mouth and teeth are kept in a healthy condition. In the press of business of attending to our patients we forget these things, and as I have before said, I keep slips containing directions near by me, so that I can hand them to patients as I discharge them. Dr. Taft has covered the ground so thoroughly, that there is very little left to be said. I commend the paper, because it tells us to do our duty rather than what our duty is.

*Dr. W. H. Whitslar*, Cleveland :—There was a discussion at a previous session of the society on the care of teeth during pregnancy and lactation, and it strikes me that this subject would have come under that head very nicely. The means of proper food, the proper filling of teeth and care of the mental state were touched upon in that discussion, but nothing, to my knowledge, was said in regard to the teeth from a hygienic standpoint. It has been my experience that women during the period of pregnancy and lactation have so much to do in taking care of children that their minds are constantly occupied in that direction, consequently they neglect their teeth. If they had taken care of them, provided they were of good structure, they would not have decayed. We must not at all times say that teeth decay because they lose their lime salts, or because they are not properly filled. Care of teeth during pregnancy and lactation by the proper means, the use of the brush, etc., will prevent them from decaying in a great many instances.

*Dr. J. Taft:*—It strikes me that this is a matter of importance both to the dentist himself and the profession, because the greater efficiency he can secure in that which he does for his patients in the way of arrest of decay of the teeth, the better will he be held in the estimation of those whom he serves. It is his duty from the stand-point of self-preservation, so far as professional reputation is concerned, and it is his duty to give such directions as will secure the most perfect results in this respect. There are various conditions in life; there is a lack of care on the part of the people in regard to their teeth. Sometimes there may have been much work done in the mouth of the patient, and he has received no instruction about its care, and by and by he or she becomes negligent and an accumulation of debris is made upon the teeth and remains for an indefinite period of time. Indeed, all of you have seen cases of the first, second and third molar, where there has been an accumulation of material upon the surface, undergoing decomposition, and you can almost predict in many instances that beneath this accumulation there is disintegration of tooth substance. If a filling has been put into the surface of the tooth you will almost instinctively come to the conclusion that the condition is faulty, and I suspect about that filling there is decay. In a great many instances it will be found that disintegration takes place there. Now then, it is important for the dentist, from a professional stand-point that he should pursue that course in the way of impressing upon the minds of his patients the necessity for care in this respect for the preservation of their teeth.

Another point. Every one, who is a regular patient; should have his or her mouth examinsd at certain intervals, perhaps every six months on an average. Some patients ought to have their teeth examined every three months. The dentist should see that the patient is careful in this matter, if possible, and not allow him or her to lapse into careless habits; he should see that they are masticating their food well, and that there are no extensive breaches made in the teeth. Other patients will go along six months without an examination of their teeth being necessary, and still others nine months, and you feel satisfied to let them go that long, knowing that they are careful in their habits. Another patient of different habits will have large decay made within a year's time. The character of the teeth, their susceptibility to disease, modify the circumstances relative to the frequency with which the teeth should be examined. The dentist who examines the teeth of regular patients should see that every filling is in good condition all the while. Filling should be gone over and kept in good condition, for there is no reason in the world why fillings may not be impaired by use in three or four years, by abrasion or roughness produced in various ways. When we get a hole in our boot, we have it repaired; when we get a hole in our coat, we have it mended; but teeth oftentimes go along without being touched. Teeth should be examined in this respect; and we should insist upon those who hold us responsible for their teeth, that these examinations shall be made. A patient holds you responsible for what you have done. We hear it said sometimes, "Dr. So and So filled my teeth, and they are all gone to pieces." Insist upon seeing the cases frequently and do whatever is necessary to maintain a perfect condition of the mouth. That is due to the dentist. As he builds up his reputation so he is established in the community. It is a duty we owe both to ourselves and patients.

